# Multi-Phase Interleaved AC–DC Step-Down Converter with Power Factor Improvement

**DOI:** 10.3390/mi14030511

**Published:** 2023-02-22

**Authors:** Jose M. Sosa-Zuniga, Christopher J. Rodriguez-Cortes, Panfilo R. Martinez-Rodriguez, Gerardo Vazquez-Guzman

**Affiliations:** 1 Tecnologico Nacional de Mexico/ITS de Irapuato, Irapuato 36821, Gto., Mexico; 2 Facultad de Ciencias, Universidad Autonoma de San Luis Potosi, San Luis Potosi 78295, SLP, Mexico

**Keywords:** PFC, rectifier, interleaved buck converter, AC–DC, LC filter

## Abstract

This paper presents the converter design of a single-phase non-isolated step-down controlled rectifier for power factor improvement and output voltage regulation. The converter consists of a full-bridge diode rectifier and a DC–DC interleaved buck converter of two or more switching cells that has an LC filter in its input. It is proposed that the interleaved switching cells operate in discontinuous conduction mode and the current through the input LC filter be continuous, avoiding switching frequency components to be injected into the grid. The controller, which has a simple structure and a small number of sensors, allows the system to achieve a high power factor. It also regulates the output voltage to a constant reference. An experimental prototype is built and tested to validate the analysis and proposed design. The closed-loop converter is evaluated both in a steady state and in transient conditions. At steady state, the converter achieves a power factor above 0.9 with a maximum of 45.4% THD at 110.1W. The main contributions of this paper are guidelines for the design of the converter, open-loop analysis, and converter control.

## 1. Introduction

Power Factor Correction (PFC) rectifiers are essential in the AC–DC conversion required to supply power to different loads. They are preferred in industrial applications for reducing the harmonic distortion of the AC current and achieving a power factor (PF) close to unity which maximizes the active power transferred from the AC grid [1]. PFC rectifiers must have sinusoidal waveform AC current, regulated DC output current or regulated DC output voltage, simple control and modulation schemes, and high efficiency [2]. They can be composed of two stages, where the first stage is responsible for the PFC, and the second is for voltage or current regulation. Single-stage PFC rectifiers can reduce the number of components and increase efficiency.

PFC rectifiers can be galvanically isolated or not, and can operate in Continuous Conduction Mode (CCM) or in Discontinuous Conduction Mode (DCM) [3], and are required in different applications and in a wide power range. For example, in domestic applications, there is a need for motor drivers that are used in ventilation, air conditioning, and dryer applications [4]. In lighting applications, they are required in LED lighting [5,6,7] and in high-pressure sodium lamps [8]. In particular, LED drivers require unidirectional rectifiers that achieve near unity PF, low switching ripple [9], and long lifetime [10]. Other applications are in drivers for induction motors or permanent magnet motors for elevators that normally use a rectifier system followed by a DC–AC conversion stage [11,12]. PFC rectifiers have applications in charging low-capacity lithium-ion batteries, such as those used in electronic devices, including mobile phones, which require high power density designs but are also needed for charging electric vehicle (EV) battery banks. Those converters used as on-board or off-board EV chargers can be unidirectional or bidirectional [13,14,15,16]. A review of single-phase unidirectional non-isolated PFC converters for onboard battery chargers can be found in [16]. Other applications are in uninterruptible power supplies for data centers [17], and wireless power transfer systems [18].

The boost converter-based PFC rectifier has been widely adopted due to its simplicity and high efficiency [19,20,21,22,23]. The converter DC output voltage in such systems is typically higher than the peak of the AC supply voltage, and for step-down applications, a second stage is required to regulate to a lower voltage level [21] which may degrade the efficiency of the system. PFC rectifiers based on conventional converters such as buck-boost [24], SEPIC [21,25], Cuk [12,25,26], flyback [27], Luo [28], and Zeta [29] converters can improve PF as well as have step-down conversion capability. Very few works have focused on the conventional buck converter since, without modification, it has a discontinuous AC input current and, consequently, high harmonic distortion [30]. However, step-down PFC rectifiers are increasingly being used to achieve a wider control range for the output voltage and to reduce the step-down requirement in the DC–DC conversion stage, for example, in EV charging systems [31]. Their use results in DC–DC converters being built with low-voltage switches, leading to higher efficiency [15]. Step-down PFC rectifier systems are expected to provide an option for supplying DC distribution grids or also for charging EV batteries [2].

In [32], a PFC AC–DC system is presented for applications of less than 100 W. The system uses a full-bridge diode rectifier, a charge pump circuit, and a class-DE resonant circuit. The class-DE topology is similar to the class-D topology but with switching conditions like the class-E circuit [33]. However, the topology in [32] suffers from increased electrical stress as a result of the addition of the charge pump circuit, which results in additional losses in the resonant tank. In [4], the control of a switched reluctance motor with a converter consisting of two Cuk converters with a common switch, in DCM and with AC supply voltage is presented. The converter includes PFC and operates in DCM, which reduces its size and cost. However, a controller is required to keep the voltage across the two capacitors of the dual converter balanced. In [26], a converter based on the switched inductor Cuk converter in CCM is presented for battery charging applications with a nominal power of 500 W. The topology has a high step-down gain and a relatively small number of components. However, the topology has relatively large inductors due to CCM operation and complex control. In [5], a driver for LEDs is proposed that consists of a first stage of a PFC rectifier and a second stage based on a bidirectional buck-boost converter. The bidirectional buck-boost converter is connected in parallel with the output of the PFC converter and serves to absorb the second harmonic component of the output current. However, control of the parallel converter can be complex, and generally, the topology has a relatively large inductor leading to large core and winding losses. In addition, the passive and active components of the parallel converter may suffer from high voltage stress. In [34], a non-linear control for a two-switch buck-boost PFC rectifier is proposed, with an active power decoupling function that can avoid the use of large electrolytic capacitors. Elements in the added circuit suffer from high voltage stress, and the converter controller is complex. A PFC rectifier without electrolytic capacitors based on the flying capacitor buck-boost converter is presented in [35]. The topology incorporates additional components, and due to the converter’s nonlinear dynamics, control design is difficult. In addition, the output voltage must be greater than half of the peak AC voltage.

This paper presents the design of a single-phase step-down PFC rectifier together with its control. The converter consists of a full-bridge diode rectifier and a DC–DC interleaved buck converter of two or more switching cells that has an LC filter at its input. The proposed control is conventional and is based on a two-loop average control that assumes decoupled voltage and current dynamics. However, in this case, the decoupling occurs naturally due to the design by proposing that the LC input filter has a continuous current in the inductor and a continuous voltage across the capacitor and that the interleaved switching cells operate in DCM. With these assumptions, the control can be configured with a simple structure with only the feedback of three variables from the converter, namely the AC grid current, the voltage across the DC output capacitor, and the grid AC voltage. In DCM operation, switching frequency harmonic components can be conducted, and active switches can withstand high voltage spikes. However, the noise conducted into the electrical grid is mitigated by the input LC filter avoiding large switching frequency components being injected into the AC grid. Additionally, the interleaved switching cells allow current and voltage ratings to be shared between each switching cell and then reduce element sizes. The main contributions of this paper are the guidelines for the design of the converter, open-loop analysis and modeling, and the proposal of the converter control.

## 2. Multi-Phase Interleaved AC–DC Step-Down Converter Description

**Figure 1:**
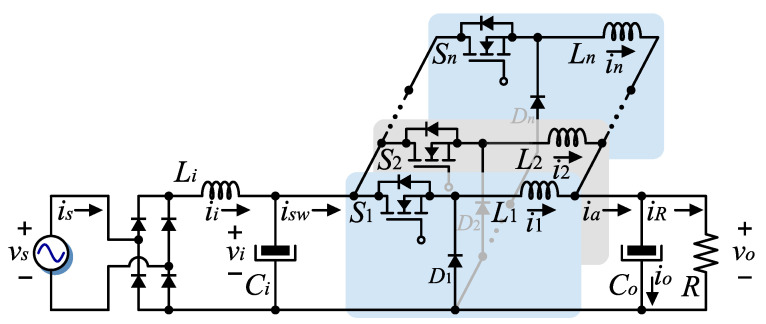
Diagram of the converter topology.

The electric simplified circuit of the converter is shown in Figure 1 and consists of a full-bridge diode rectifier, and a DC–DC interleaved buck converter, which has an LC filter at its input, formed by the inductor $L_{i}$ and capacitor $C_{i}$, which is connected to a buck converter of *n* interleaved switching cells, with n≥2. It is proposed that the interleaved switching cells operate in DCM and also that the current through the input filter be continuous, avoiding large switching frequency components being injected into the AC grid.

The buck converter has *n* active switches $S_{1},\ldots ,S_{n}$, *n* diodes $D_{1},\ldots ,D_{n}$, an output DC capacitor $C_{o}$, and *n* inductors $L_{1},\ldots ,L_{n}$ for the interleaved switching cells. The load is represented by the resistive element *R*, and the AC electrical grid is represented by a voltage source. The converter operation in steady state is set such that the currents through the inductors $L_{1},\ldots ,L_{n}$ are in DCM, and the current through $L_{i}$, $i_{i}$, and the voltage across $C_{i}$, $v_{i}$, are in CCM. The switches $S_{l}$, with l=1,…,n, are switched at a frequency f=1/T where *T* is the switching period in seconds. The activation and deactivation pattern of each $S_{l},\;l=1,\ldots ,n$ is equal but is displaced T/n s, consecutively and cumulatively. Let $k:=t_{\mathrm{on}}/T$ denote the duty cycle where $t_{\mathrm{on}}$ is the time when the active switches remain in conduction. Compared to the converter with only one single switching cell in DCM, the incorporation of *n* interleaved switching cells $\left\{S_{l},L_{l},D_{l}\right\}$, with l=1,…,n>1, reduces the electrical stress on the switching devices, leading to the use of smaller size elements as well as reducing current ripple in the load. The input filter performs a low-pass filter function that mitigates the switching harmonic components injected into the AC grid. This input filter can be designed to ensure a continuous grid current and continuous capacitor voltage $v_{i}$, both with reduced ripple. Under this condition, and in steady state, $v_{i}$ can be assumed constant except for the voltage ripple, and therefore the input filter dynamics can be supposed to be decoupled from the output DC dynamics with the objective to simplify the output voltage regulation control design.

## 3. Converter Steady-State Analysis

In this section, the analysis of the open-loop steady-state operation of the converter is presented. The assumptions considered in the analysis are the following.

1. All elements, passive and active, are ideal. In particular, parasitic series resistances of inductors are not considered.
2. Inductors $L_{1}=\cdots =L_{n}=L_{o}$ are equal.
3. Duty cycles and phase-shift angles are equal for each interleaved switching cell $\left\{S_{l},L_{l},D_{l}\right\}$, l=1,…,n.

The analysis of the converter with a DC voltage source in the next subsection establishes design guidelines so that the current in each of the interleaved inductors is discontinuous and input current $i_{i}$ and voltage $v_{i}$ are continuous. Therefore, this analysis yields rules for the selection of:

1. The inductor value $L_{o}$ to assure DCM in terms of load and switching frequency,
2. the values of $L_{i}$ and $C_{i}$ to assure a constant positive current $i_{i}$ and constant positive voltage $v_{i}$ with small ripple, and,
3. the output capacitor value $C_{o}$ to assure a given output voltage ripple in terms of the load.

In the analysis of the converter connected to an AC voltage source through a full-bridge diode rectifier is supposed that conditions of the operation previously described are preserved.

### 3.1. Steady-State Analysis with a DC Input Voltage Source

**Figure 2:**
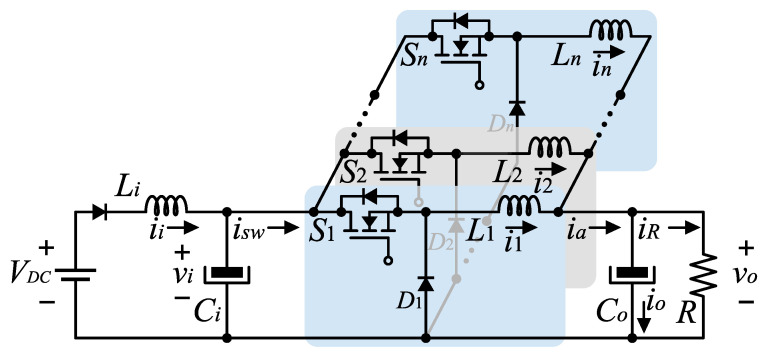
Converter topology with a constant DC power supply.

The full-bridge diode rectifier and the AC voltage source in Figure 1 are replaced by a constant DC voltage source with value $V_{DC}$, as shown in the circuit of Figure 2.

The notation of the variables is as follows. Let $i_{l}$ with l=1,…,n be the instantaneous current through the inductor $L_{l}$, let $i_{R}$ be the instantaneous current through the load, and let $v_{i}$ and $v_{o}$ denote the instantaneous voltages through the capacitors $C_{i}$ and $C_{o}$, respectively.

**Figure 3:**
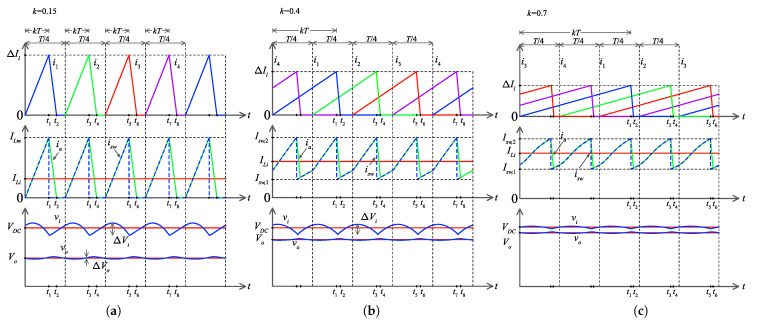
Converter steady-state waveforms under different duty cycles *k*, (**a**) 0.15, (**b**) 0.4, (**c**) 0.7.

In Figure 3, the waveforms of the converter with *n* = 4 interleaved cells are depicted for three different duty cycles. The general case for *n* interleaved switching cells is analogous. In Figure 3a, k=0.15, in Figure 3b, k=0.4, and in Figure 3c, k=0.7. The upper plots depict the currents $i_{l}$, l=1,…,n of the interleaved inductors, the middle plot depicts the sum of the switch currents $i_{sw}$ and the sum of inductor currents $i_{a}=i_{1}+\cdots +i_{n}$. The plot at the bottom depicts the voltage of capacitors $v_{i}$ and $v_{o}$. With duty cycles less than 1/n, the currents $i_{l}$, l=1,…,n do not overlap and are zero before any other rises.

#### 3.1.1. Selection of $L_{o}$

By assuming that $v_{i}$ is positive and continuous, then its averaged value is $V_{i}=V_{DC}$ in the steady state. Therefore, the converter can be simplified as an interleaved buck of *n* switching cells connected to a constant voltage source. From the circuit $i_{a}=\sum_{l=1}^{n}i_{l}=i_{o}+i_{R}$ and then $I_{a}=I_{R}=V_{o}/R$, where uppercase denotes DC component. Therefore $I_{l}=V_{o}/\left(nR\right)$, and the current peak of interleaved inductors is given by

(1) $$\begin{array}{c} \Delta I_{l}=\frac{(V_{DC}-V_{o})k}{L_{l}f},l=1,\ldots ,n. \end{array}$$

Then the critical inductor for DCM is given by $2I_{l}=\Delta I_{l}$, and the critical inductor for the interleaved inductors to achieve DCM is given by the following expression

(2) $$\begin{array}{c} L_{o,c}=\frac{(V_{DC}-V_{o})knR}{2fV_{o}}=\frac{(1-k)nR}{2f}. \end{array}$$

Then, the inductor $L_{l}<L_{o,c}$ to assure DCM of the switching cells, which implies that the current $i_{l},l=1,\ldots ,n$ is zero from a given time on during a switching period. Additionally, the static output voltage is given by,

(3) $$V_{o}=\frac{2V_{DC}}{1+\sqrt{1+\frac{8L_{e}f}{Rk^{2}}}}=\frac{2V_{DC}}{1+\sqrt{1+\frac{8L_{o}f}{nRk^{2}}}},$$

where the equivalent inductor $L_{e}=L_{o}/n$ is given by the parallel connection of $L_{1},\ldots ,L_{n}$.

#### 3.1.2. Selection of $L_{i}$ and $C_{i}$

**Figure 4:**
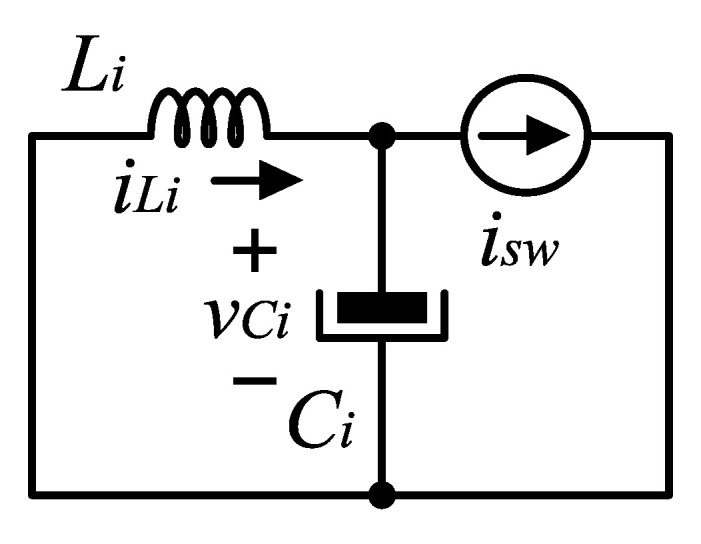
Simplification of the input filter circuit.

The passive elements $L_{i}$ and $C_{i}$ are selected to assure a constant positive current $i_{i}$ and constant positive voltage $v_{i}$ with a small ripple. The objective of this input filter is to reduce the switching frequency harmonics in $i_{i}$. Its cutoff frequency is selected relatively low, and the current $i_{sw}$ can be treated as an input that excites, in steady state, the current $i_{i}$ as depicted in Figure 4. The largest amplitude component of $i_{i}$ has the natural frequency of the input filter $1/\sqrt{L_{i}C_{i}}$ rad/s. This frequency has to be selected large enough to avoid resonance problems with grid frequency harmonics, but less than the switching frequency to filter effectively.

The analysis proceeds by obtaining the steady-state expression of $i_{i}$ excited by $i_{sw}$, by discarding the DC component. In the steady state, and for duty-cycle k<1/n the current $i_{sw}$ has the larger ripple amplitude, is periodic with period T/n, and can be described by

(4) $$i_{sw}\left(t\right)=\left\{\begin{array}{cc} \frac{I_{m}t}{kT}, & 0<t\leq kT,\\ 0, & kT<t\leq T/n, \end{array}\right.$$

where $I_{m}$ is the peak amplitude of any of the inductor currents $i_{l},l=1,\ldots ,n$, and can be obtained by (1). Hence, the average is given by $I_{sw}=nkI_{m}/2$, and the zero average form of $i_{sw}$, $i_{sw,ac}$ is

(5) $$i_{sw,ac}\left(t\right)=\left\{\begin{array}{cc} \frac{I_{m}t}{kT}-\frac{nkI_{m}}{2}, & 0\leq t\leq kT,\\ -\frac{nkI_{m}}{2}, & kT\leq t\leq T/n. \end{array}\right.$$

For analysis, current $i_{sw,ac}$ can be approximated by a sinusoidal of period T/n, with the same RMS value than $i_{sw,ac}\left(t\right)$

(6) $$i_{sw,ac,\mathrm{RMS}}=\frac{I_{m}}{6}\sqrt{3nk(4-3kn)}.$$

Then

(7) $$i_{sw,ac}\left(t\right)\approx i_{\sin}\left(t\right)=\frac{I_{m}}{6}\sqrt{6nk(4-3kn)}\sin\left(2\pi nt/T\right).$$

The equations of the circuit in the Figure 4 are given by

(8) $$L_{i}\;di_{i}/dt=-v_{i},$$

(9) $$C_{i}\;dv_{i}/dt=i_{i}-i_{\sin}(t).$$

The solution is periodic and has two frequency components, one at *n* times the switching frequency and the other at the resonance frequency of the input filter $1/\sqrt{L_{i}C_{i}}$. The latter has the larger amplitude, and by ignoring the switching frequency component, a solution is given by

(10) $$i_{i}\left(t\right)\approx \frac{n\pi TI_{m}\sqrt{6nkL_{i}C_{i}\left(4-3nk\right)}}{3(4n^{2}\pi^{2}L_{i}C_{i}-T^{2})}\sin\left(t/\sqrt{L_{i}C_{i}}\right),$$

(11) $$v_{i}\left(t\right)\approx \frac{-n\pi L_{i}TI_{m}\sqrt{6nk\left(4-3nk\right)}}{3(4\pi^{2}L_{i}C_{i}n^{2}-T^{2})}\cos\left(t/\sqrt{L_{i}C_{i}}\right).$$

Therefore an approximation for the current ripple can be given by

(12) $$\Delta I_{i}\approx \frac{2n\pi TI_{m}\sqrt{6nkL_{i}C_{i}\left(4-3nk\right)}}{3(4n^{2}\pi^{2}L_{i}C_{i}-T^{2})}$$

(13) $$\Delta V_{i}\approx \frac{2n\pi L_{i}TI_{m}\sqrt{6nk\left(4-3nk\right)}}{3(4\pi^{2}L_{i}C_{i}n^{2}-T^{2})}$$

The averaged values of the inductor current and capacitor voltage are given by $I_{i}=knI_{m}/2$ and $V_{i}=V_{DC}$, then the selection of passive elements, for assuring continuous current and voltage, must follow

(14) $$\Delta I_{i}<2I_{i}=knI_{m},\;\Delta V_{i}<2V_{i}=2V_{DC}.$$

However, in practice, and to reinforce the decoupling between input and output dynamics, $\Delta V_{i}$ must be selected small. By design, current $i_{i}$ and voltage $v_{i}$ in steady-state are continuous, and their ripples have a fundamental frequency of $1/\sqrt{L_{I}C_{I}}$, which is much less than the switching frequency. The input filter’s natural frequency must be larger than any of the expected harmonic components in the AC voltage source.

#### 3.1.3. Selection of $C_{o}$

The output capacitor $C_{o}$ must filter the sum of currents of the interleaved inductors $i_{a}$ to obtain a continuous voltage in the load. In the worst case scenario, for kT≤T/n, $i_{a}$ is discontinuous and $C_{o}$ can be computed by requiring a given $\Delta V_{o}$ per [36],

(15) $$C_{o}=\frac{T\left(nk+d_{2}\right){\left(I_{m}-I_{o}\right)}^{2}}{2nI_{m}\Delta V_{o}},$$

and where $d_{2}$ is the fraction of T/n where the $i_{l}$ drops to zero whenever k≤T/n and is given by,

(16) $$d_{2}=\frac{-nk+\sqrt{n^{2}k^{2}+\frac{8nL_{o}}{RT}}}{2}.$$

## 4. Controller Design for the Converter with a Full-Bridge Rectified Sinusoidal Power Supply

In this subsection, the controller design and arguments for the performance of the closed-loop system are presented. The converter of the previous subsection is considered, but by replacing the DC voltage source with a full-bridge diode rectified AC voltage source.The following assumptions are made.

1. The AC grid is considered without harmonic distortion and is represented by $v_{s}\left(t\right)=V_{m}\sin\left(\omega_{s}t\right)$, where $V_{m}$ is the peak voltage in Volts (V) and $\omega_{s}$ is the constant grid angular frequency in rad/s.
2. Switching frequency $2\pi f_{sw}$ is much higher than the grid angular frequency $\omega_{s}$ so that the input voltage can be considered constant during one switching period.
3. Inductor currents $i_{l}$, l=1,…,n are discontinuous in the steady state.

A complete standard state equation model is essentially nonlinear and is difficult to obtain due to the DCM nature of the output cells, in addition to the rectification stage. Although the converter has multiple switching cells that operate in DCM, its dynamics can be approximated by a model that describes the variables averaged in a switching frequency period. To simplify, the interleaved buck is replaced by an equivalent single-cell buck converter in DCM that has the equivalent inductor $L_{o}/n$. Moreover, to avoid considering the derivative of the rectified voltage, $|v_{s}\left(t\right)|$, which is not well defined in the zero-crossings of $v_{s}$, the grid current $i_{s}$ dynamics is considered instead of the dynamics of $i_{i}$. Therefore, a state-space model that can approximate the converter dynamics is given as:

(17) $$L_{i}{\dot{x}}_{1}=-x_{2}+v_{s}\left(t\right),$$

(18) $$C_{i}{\dot{x}}_{2}=x_{1}-u_{s}x_{3},$$

(19) $$\left(L_{o}/n\right)\;{\dot{x}}_{3}=-dx_{4}+u\left(x_{2}-x_{4}\right),$$

(20) $$C_{o}{\dot{x}}_{4}=dx_{3}-\frac{1}{R}x_{4}+ux_{3}.$$

The variable $x_{1}$ denotes the grid current $i_{s}$, and therefore $|x_{1}|$ represents the averaged current $i_{i}$. Likewise, $|x_{2}|$ represents the averaged capacitor voltage $v_{i}$, and $x_{3}$ and $x_{4}$ are averaged variables in a switching frequency period *T* that are related to $i_{a}$ and $v_{o}$, respectively. The variable u∈(0,1) is the duty-cycle and is considered the control input, and $u=|u_{s}|$. On the other side, *d* denotes the fraction of *T* that takes the current of the equivalent inductor $L_{o}/n$ to drop to zero in the DCM buck converter. In this analysis, it is considered that *d* is an unknown constant, but satisfying d<u. In general, *d* depends on *R*, *u*, $v_{s}$ and $L_{o}$. Equations (17)–(20) are non-linear and may describe the averaged behavior of the converter. The control objectives are the following

1. Current tracking: $i_{s}\to Gv_{s}\left(t\right)$, as t→∞, where *G* is a positive constant.
2. Voltage regulation: $v_{o}\to V_{ref}$ as t→∞, where $V_{ref}$ is a positive constant.

The proposed control is conventional and is based on a two-loop averaged control that assumes decoupled voltage and current dynamics. By following the design of the previous section, the input dynamics are assumed much faster than the output dynamics, and therefore, input and output dynamics can be considered decoupled. Therefore, the control objectives of output voltage regulation to constant references and AC input current tracking can be designed independently, and the control can be configured with a simple structure and a reduced number of sensors, namely the grid current, the grid voltage, and the voltage across the DC output capacitor. The inner current control loop forces the grid current to follow as closely as possible a sinusoidal reference that is proportional to the fundamental component of the input AC voltage. The outer voltage control loop regulates the average output voltage.

### 4.1. Current Tracking Control Loop

The control proposal proceeds by defining the current tracking loop. Since the current tracking objective imposes $i_{s}$ to be proportional to $v_{s}$ then it is proposed that $u_{s}=k_{c}\left(i_{s}^{*}-i_{s}\right)=k_{c}\left(gV_{m}\sin\left(\omega_{s}t\right)-x_{1}\right)$ where *g* is to be defined in the voltage regulation loop. Then the input filter dynamics become

$$\begin{array}{ccc} L_{i}{\dot{x}}_{1} & = & -x_{2}+V_{m}\sin\left(\omega_{s}t\right),\\ C_{i}{\dot{x}}_{2} & = & x_{1}-k_{c}\left(gV_{m}\sin\left(\omega_{s}t\right)-x_{1}\right)x_{3}. \end{array}$$

Per the nature of the converter, $x_{3}$ is always positive, and by assuming it is constant, we can obtain the steady-state response by,

(21) $$x_{1}^{*}=\left(\frac{K_{c}}{1+K_{c}}\right)gV_{m}\sin\left(\omega_{s}t\right)$$

(22) $$x_{2}^{*}=V_{m}\sin\left(\omega_{s}t\right),$$

where $K_{c}=k_{c}x_{3}$. It can be observed that $x_{1}^{*}\left(t\right)\approx gV_{m}\sin\left(\omega_{s}t\right)$ whenever $K_{c}\gg 1$. The error dynamics, with error state variables $z_{1}=x_{1}-x_{1}^{*}$ and $z_{2}=x_{2}-x_{2}^{*}$ is given by

$$\begin{array}{ccc} L_{i}{\dot{z}}_{1} & = & -z_{2}-\left(\frac{K_{c}}{1+K_{c}}\right)gL_{i}V_{m}\cos\left(\omega_{s}t\right)\\ C_{i}{\dot{z}}_{2} & = & \left(1+K_{c}\right)z_{1}-C_{i}\omega_{s}V_{m}\cos\left(\omega_{s}t\right). \end{array}$$

The latter is a forced harmonic oscillator whose solutions have a transient with natural frequency oscillation and, in steady state, have the response given by (21) and (22).

### 4.2. Output Voltage Regulation Control Loop

Given that $i_{l}$, l=1,…,n are discontinuous at the switching frequency, therefore the voltage $v_{i}=V_{in}$ can be assumed constant. The output dynamics are given by

(23) $$\left(L_{o}/n\right){\dot{x}}_{3}=-dx_{4}+g\left(V_{in}-x_{4}\right)$$

(24) $$C_{o}{\dot{x}}_{4}=dx_{3}-\frac{1}{R}x_{4}+gx_{3}$$

and therefore, to achieve output voltage regulation, a PI controller is proposed

(25) $$g=k_{p}\left(V_{ref}-v_{o}\right)+k_{i}\eta$$

(26) $$\dot{\eta }=V_{ref}-v_{o},$$

where $V_{ref}$ is the constant reference for the output voltage. Therefore the equilibrium point for the output dynamics in a closed loop is given by

(27) $$x_{3}^{*}=\frac{V_{ref}\left(V_{in}-V_{ref}\right)}{V_{in}dR},\;x_{4}^{*}=V_{ref},\;\eta^{*}=\frac{dV_{ref}}{k_{i}\left(V_{in}-V_{ref}\right)},$$

where $V_{in}$ is the voltage $v_{i}$ assumed constant with a low ripple. By defining error variables $z_{3}:=x_{3}-x_{3}^{*}$, $z_{4}:=x_{4}-x_{4}^{*}$ and $z_{5}:=\eta -\eta^{*}$, and considering the linearized system we obtain,

(28) $$\left(L_{o}/n\right){\dot{z}}_{3}=-\left(k_{p}\left(V_{in}-V_{ref}\right)+\left(\frac{dV_{in}}{V_{in}-V_{ref}}\right)\right)z_{4}+k_{i}\left(V_{in}-V_{ref}\right)z_{5},$$

(29) $$C_{o}{\dot{z}}_{4}=\left(\frac{dV_{in}}{V_{in}-V_{ref}}\right)z_{3}-\left(k_{p}x_{3}^{*}+1/R\right)z_{4}+k_{i}x_{3}^{*}z_{5},$$

(30) $${\dot{z}}_{5}=-z_{4}.$$

**Figure 5:**
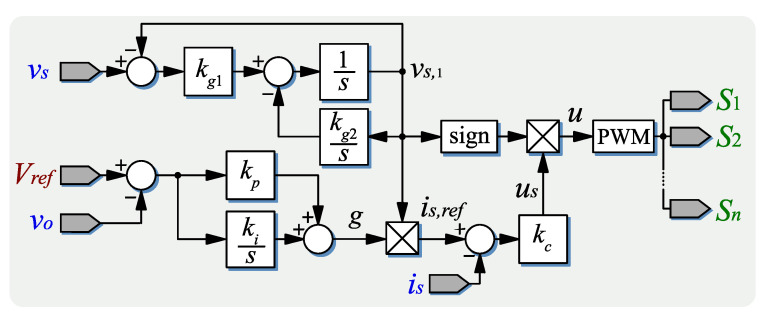
Diagram of the proposed controller.

Therefore $k_{p}$, $k_{i}$ can be computed numerically to obtain a given closed-loop transient response locally. The complete proposed controller is shown in the block diagram of Figure 5.

In order to cope with possible distortion in the grid voltage, an estimator of the fundamental component $v_{s,1}$ is implemented as in [37], where $k_{g1}>0$ is a constant that is related to the velocity of the convergence of the estimation, and $k_{g2}=\omega_{s}^{2}$. Then, $v_{s,1}$ is used in the controller instead of the possibly distorted $v_{s}$.

It is worth noting that although the design of the output dynamic control proceeds by assuming constants, the $v_{i}$ voltage has a second harmonic grid frequency component, and therefore, the output voltage regulation is performed in average. The guidelines for converter parameter selection are presented in Section 3. These result in a range of converter parameters for which the closed-loop operation will produce the expected results. As long as the current of $L_{l}$ remains discontinuous in the steady state, and the current of $L_{i}$ and the voltage of $C_{i}$ remain continuous, the operation of the converter will vary qualitatively little in open loop, and the conditions for the controller will be preserved. Regarding the control parameters, all the gains are positive and do not directly depend on the converter parameters. Then, slightly modifying the controller gains is expected to slightly modify the closed-loop transient response, such as damping, overshoot, and settling time.

## 5. Experimental Results

Experimental tests are carried out to verify the performance of the closed-loop system in a laboratory prototype with n=4 interleaved cells. The fine adjustment of the converter and control parameters is established by means of numerical simulations. For the converter parameters, the general rules described previously are followed. For example, the current of each inductor of the interleaved cells is discontinuous, which is ensured by setting $L_{1},L_{2},L_{3}$, and $L_{4}$ less than $L_{o,c}$ in (2). In addition, the current of $L_{I}$ and the voltage of $C_{i}$ are continuous at steady state and are selected according to (14). Regarding the control parameters, all the gains are positive and do not directly depend on the converter parameters. However, some general rules can also be followed, for example, $k_{c}$ is chosen to be greater than $k_{p}$ to force decoupling between input current and output voltage dynamics. The higher the gain $k_{i}$, the faster the convergence to the output voltage reference during voltage reference transitions or load changes. However, overshoot and oscillations increase, and a large value will lead to instability. In the estimation of the fundamental component, $k_{g2}$ must be equal to $\omega_{s}^{2}$, and $k_{g1}$ is only required to be positive. The gain $k_{g1}$ has an effect only at startup and is related to the speed of convergence for the generation of the current reference. The higher the gain $k_{g1}$ is, the faster the convergence will be in the generation of the current reference $i_{s,ref}$ without any stability issue.

**Figure 6:**
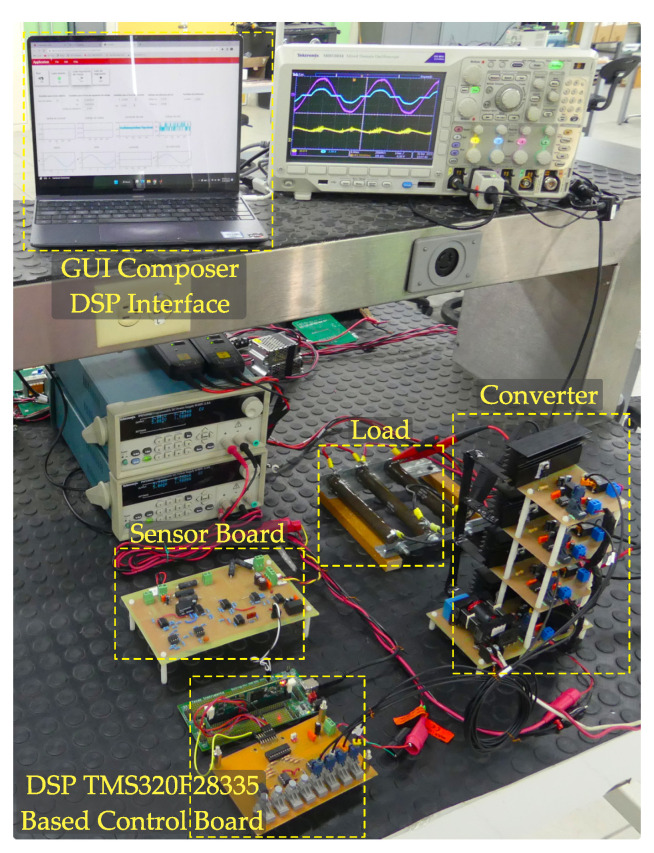
Experimental setup of the proposed multi-phase interleaved AC–DC step-down converter with power factor improvement.

The parameters of the converter are shown in Table 1, and the experimental prototype is shown in Figure 6. The controller is implemented using the digital signal processor DSP TMS320F28335, and the switching signals are generated with the ePWM modules c2833x of the same DSP circuit. For the active switches, the CoolMOS${}^{\mathrm{TM}}$ transistor SPP20N60C3 is used; for the output inductors, the power inductor 60A363C from Murata is used; and, for the freewheeling diodes, the SiC diode IDD10SG60C is utilized.

### 5.1. Steady-State Response

In this subsection, the closed-loop responses in the steady state due to two different output voltage references are presented. The reference $V_{ref}=60$ V represents a power of 49.3 W, and the reference $V_{ref}=90$ V represents a power of 110.1 W.

**Figure 7:**
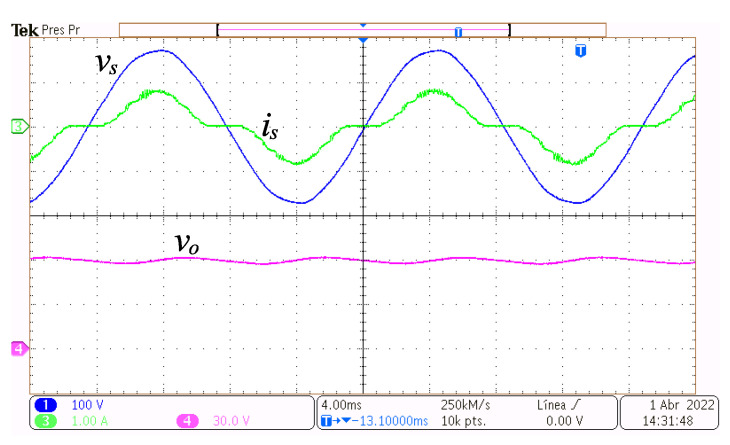
Steady-state operation at 49.3 W. Grid voltage $v_{s}$ (y-axis 100 V/div, x-axis 4 ms/div), grid current $i_{s}$ (y-axis 1 A/div, x-axis 4 ms/div), and output voltage $v_{o}$ (y-axis 30 V/div, x-axis 4 ms/div).

In Figure 7, at the top, the grid voltage $v_{s}$ and the grid current $i_{s}$ are depicted, and at the bottom, the output voltage $v_{o}$, with the converter functioning at a power of 49.3 W. It can be observed that every depicted waveform is continuous and has a very low switching ripple. Moreover, current $i_{s}$ is in phase with $v_{s}$, although there are non-conduction intervals around the crossing of $v_{s}$ waveform with zero volts. This is because if the grid voltage is less than the output voltage at any time, then the grid is not supplying power at that time. The output voltage $v_{o}$ is almost constant at the required voltage reference $V_{ref}$ with small amplitude oscillations whose main frequency is double the grid frequency due to the rectification process. The amplitude of these oscillations is related to the value of the capacitor $C_{o}$ and the magnitude of the current $i_{a}$.

**Figure 8:**
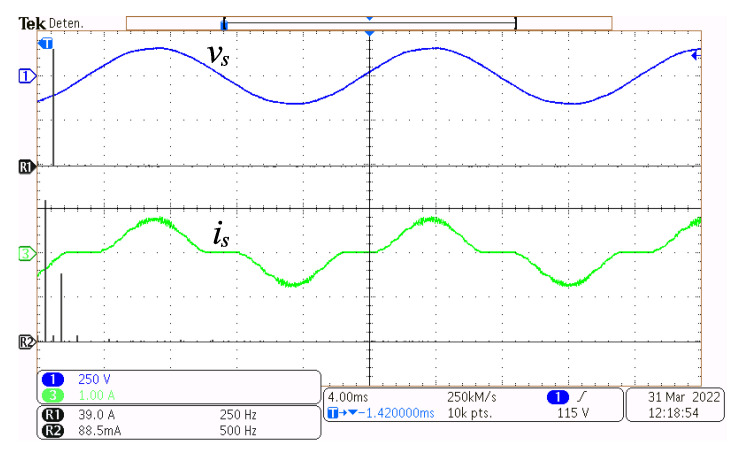
Steady-state operation at 49.3 W. Grid voltage $v_{s}$ (y-axis 250 V/div, x-axis 4 ms/div), its FFT ${v_{s}}_{\mathrm{FFT}}$ (x-axis 250 Hz), grid current $i_{s}$ (y-axis 1 A/div, x-axis 4 ms/div), and its FFT ${i_{s}}_{\mathrm{FFT}}$ (x-axis 500 Hz).

In Figure 8, the grid voltage $v_{s}$ and its Fast-Fourier-Transform (FFT) are depicted at the top, and the grid current $i_{s}$ and its FFT, are depicted at the bottom, with the converter functioning at a power of 49.3 W. It can be seen that the grid voltage is almost a clean sinusoidal by having fundamental components only at the grid frequency. The grid frequency fundamental component of current $i_{s}$ is the largest magnitude harmonic component; however, other components appear due to the distortion caused by the non-conduction time intervals.

**Figure 9:**
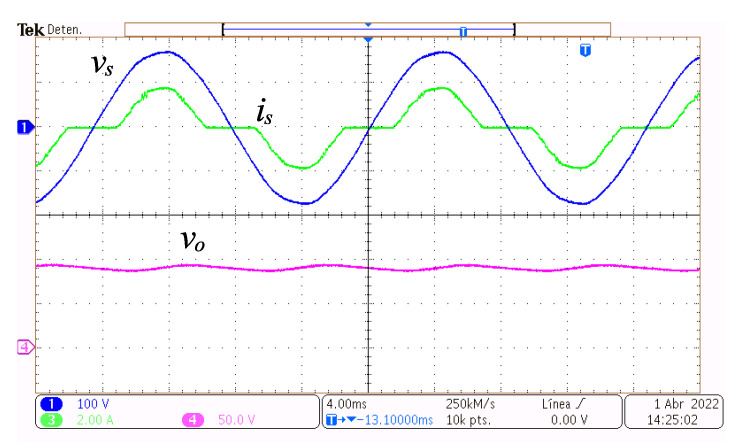
Steady-state operation at 110.1 W. Grid voltage $v_{s}$ (y-axis 100 V/div, x-axis 4 ms/div), grid current $i_{s}$ (y-axis 2 A/div, x-axis 4 ms/div), and output voltage $v_{o}$ (y-axis 50 V/div, x-axis 4 ms/div).

In Figure 9, at the top, the grid voltage $v_{s}$ and the grid current $i_{s}$ are depicted, and at the bottom, the output voltage $v_{o}$, with the converter functioning at a power of 110.1 W. In contrast to when it functions at a lower power, as depicted in Figure 9, the current $i_{s}$ has a larger amplitude, and the non-conduction intervals have increased. Nonetheless, $v_{s},i_{s}$ and $v_{o}$ are continuous and have very low switching ripple. Additionally, $i_{s}$ is in phase with $v_{s}$. The voltage $v_{o}$ is almost constant at the required voltage reference $V_{ref}=90$ V with small amplitude oscillations due to the rectification process.

**Figure 10:**
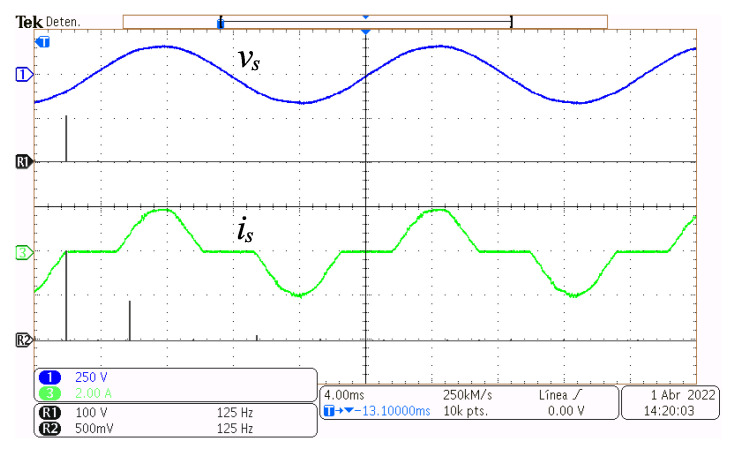
Steady-state operation at 110.1 W. Grid voltage $v_{s}$ (y-axis 250 V/div, x-axis 4 ms/div), its FFT ${v_{s}}_{\mathrm{FFT}}$ (x-axis 125 Hz), grid current $i_{s}$ (y-axis 2 A/div, x-axis 4 ms/div), and its FFT ${i_{s}}_{\mathrm{FFT}}$ (x-axis 125 Hz).

In Figure 10, the grid voltage $v_{s}$ and its Fast-Fourier-Transform, FFT, are depicted at the top, and the grid current $i_{s}$ and its FFT, are depicted at the bottom, with the converter functioning at a power of 110.1 W. The same observations can be done as with Figure 8; however, current amplitude, as well as harmonic component amplitudes, have increased.

**Figure 11:**
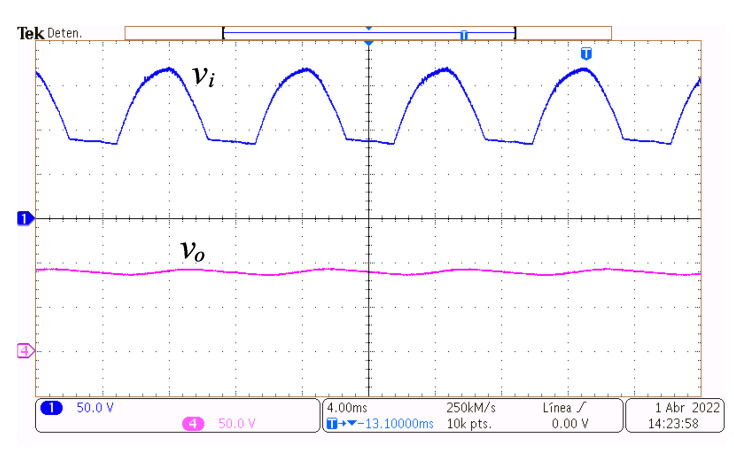
Steady-state operation at 110.1 W. Input capacitor voltage $v_{i}$ (y-axis 50 V/div, x-axis 4 ms/div), and output voltage $v_{o}$ (y-axis 50 V/div, x-axis 4 ms/div).

The Figure 11 presents the capacitors voltages $v_{i}$ and $v_{o}$ at a reference of $V_{ref}=90$ V. The voltage $v_{i}$ is shown at the top of the figure, and its waveform has a fundamental frequency twice the grid frequency. During the non-conduction intervals, $v_{i}$, on average, equals the output voltage $v_{o}$. The voltage $v_{i}$ is decreasing during the non-conduction intervals. Apart from the non-conduction intervals, $C_{i}$ is charged, and its voltage is approximately the rectified AC source voltage. At the bottom of the figure, $v_{o}$ is depicted.

The Table 2 summarizes the steady-state power quality parameters for the two different powers tested.

### 5.2. Transient Response

In this section, the experimental transient responses under step-like changes of the output voltage reference and load resistor are presented.

**Figure 12:**
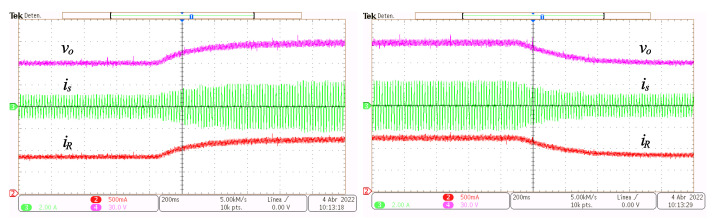
Transient response to step reference voltage changes. Output voltage $v_{o}$ (y-axis 30 V/div, x-axis 200 ms/div), grid current $i_{s}$ (y-axis 2 A/div, x-axis 200 ms/div) and load current $i_{R}$ (y-axis 500 mA/div, x-axis 200 ms/div).

In Figure 12, from top to bottom, the output voltage $v_{o}$, the grid current $i_{s}$ and the load current $i_{R}$ transient responses are depicted. The transient is caused by step-like changes of $V_{ref}$ from 60 V to 90 V and back.

**Figure 13:**
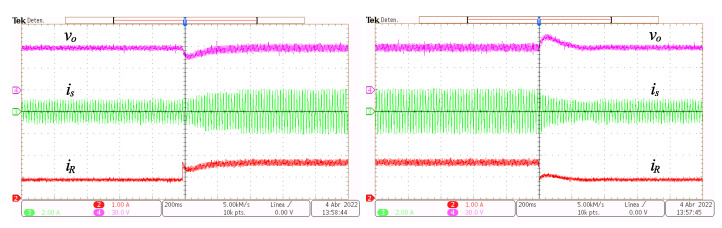
Transient response to step load changes. Output voltage $v_{o}$ (y-axis 30 V/div, x-axis 200 ms/div), grid current $i_{s}$ (y-axis 2 A/div, x-axis 200 ms/div), and load current $i_{R}$ (y-axis 1 A/div, x-axis 200 ms/div).

In Figure 13, from top to bottom, the output voltage $v_{o}$, the grid current $i_{s}$ and the load current $i_{R}$ transient responses are depicted. The voltage referenced is set constant $V_{ref}=60$ V while the load resistor is changed from 73Ω to 48Ω and back.

## 6. Conclusions

The converter design of a single-phase step-down rectifier with PFC capabilities that is based on an interleaved buck converter together with its control has been presented. The converter achieved power factor improvement on the AC power supply while at the same time being able to maintain a lower regulated DC output voltage relative to the peak AC input voltage. The proposed controller, with a simple structure, a reduced number of sensors, and a single independent switching signal for the converter, achieved the objectives of AC current tracking and DC voltage regulation. Given the proposal for interleaved operation, the size of the output filter has been reduced with components of lower current and voltage ratings compared to components of a single switching cell in discontinuous conduction mode. High-frequency conducted noise produced using discontinuous conduction mode operation that can be injected into the grid is mitigated by the input LC filter. The high voltage spikes that withstand semiconductor devices during hard switching were reduced for a given converter power because the total current and voltage ratings were shared between each interleaved switching cell. An experimental prototype with four switching cells was built and tested to validate the proposed converter and controller. The closed-loop converter was evaluated both in steady state and in transient conditions. At steady state, the converter achieved a power factor above 0.9 with a maximum of 45.4% THD at 110.1 W. The relatively high total harmonic distortion was due to the fact that the converter was based on the buck topology, and when the required output voltage was less than the value of the grid voltage, the grid current was zero, which led to periods of non-conduction around the zero crossings of the grid voltage. Thus, harmonic distortion was reduced when lower output voltages were required. The contributions of the work were, on the one hand, the presentation of the analysis of the converter operating in discontinuous conduction mode, which allows for obtaining the design parameters of the converter. On the other hand, analysis and steady-state operation waveforms were presented. Another contribution was the controller that addresses the regulation of the DC output voltage and the tracking of the input current to a sinusoidal. The dynamics of the input current and output voltage were considered to be naturally decoupled due to the proposed operation; thus, the controller achieved the objectives using the feedback of only three variables, namely the AC grid voltage, the AC grid current, and the DC output voltage. Interleaved operation provides redundancy to the converter, and closed-loop operation can be expected to achieve control objectives under open-circuit faults in the interleaved switching cell semiconductors, as long as the current or voltage of the semiconductors does not exceed their safe and reliable operating limits. Therefore, in future work, the fault-tolerance capabilities of the closed-loop converter can be experimentally investigated, improved, and evaluated. The solution has potential applications in any system that contains a rectification stage, which is required to reduce voltage level and power factor improvement, for example, in battery charging, LED-based lighting, and as a DC power source for electronic equipment.

## Figures and Tables

**Table 1:** Converter parameters.

Parameter	Value
Input Voltage	$127\;V_{\mathrm{RMS}}$
Nominal Load *R*	73 Ω
Switching Frequency $f_{sw}$	50 kHz
Grid angular frequency $\omega_{s}$	120π rad/s
Nominal Output Voltage $V_{ref}$	60 V
Input Inductor $L_{i}$	500 μH
Interleaved Inductors $L_{1},L_{2},L_{3},L_{4}$	36 μH
Input Capacitor $C_{i}$	0.47 μF
Output Capacitor $C_{o}$	820 μF

**Table 2:** Power quality parameters.

	49.3 W	110.1 W
Displacement Power Factor DPF	1	1
Power Factor PF	0.94	0.91
Input Current THD	35.9%	45.4%

## Data Availability

Not applicable.
